# Increased 4R tau expression and behavioural changes in a novel *MAPT-N296H* genomic mouse model of tauopathy

**DOI:** 10.1038/srep43198

**Published:** 2017-02-24

**Authors:** Heike J. Wobst, Franziska Denk, Peter L. Oliver, Achilleas Livieratos, Tonya N. Taylor, Maria H. Knudsen, Nora Bengoa-Vergniory, David Bannerman, Richard Wade-Martins

**Affiliations:** 1 Department of Physiology, Anatomy and Genetics, University of Oxford, Oxford, United Kingdom; 2 MRC Functional Genomics Unit, Department of Physiology, Anatomy and Genetics, University of Oxford, Oxford, United Kingdom; 3 Department of Experimental Psychology, University of Oxford, Oxford, United Kingdom; 4 Oxford Parkinson’s Disease Centre, University of Oxford, Oxford, United Kingdom

## Abstract

The microtubule-associated protein tau is implicated in various neurodegenerative diseases including Alzheimer’s disease, progressive supranuclear palsy and corticobasal degeneration, which are characterized by intracellular accumulation of hyperphosphorylated tau. Mutations in the tau gene *MAPT* cause frontotemporal dementia with parkinsonism linked to chromosome 17 (FTDP-17). In the human central nervous system, six tau isoforms are expressed, and imbalances in tau isoform ratios are associated with pathology. To date, few animal models of tauopathy allow for the potential influence of these protein isoforms, relying instead on cDNA-based transgene expression. Using the P1-derived artificial chromosome (PAC) technology, we created mouse lines expressing all six tau isoforms from the human *MAPT* locus, harbouring either the wild-type sequence or the disease-associated N296H mutation on an endogenous *Mapt*−/− background. Animals expressing N296H mutant tau recapitulated early key features of tauopathic disease, including a tau isoform imbalance and tau hyperphosphorylation in the absence of somatodendritic tau inclusions. Furthermore, N296H animals displayed behavioural anomalies such as hyperactivity, increased time in the open arms of the elevated plus maze and increased immobility during the tail suspension test. The mouse models described provide an excellent model to study the function of wild-type or mutant tau in a highly physiological setting.

Tauopathies are a group of neurodegenerative diseases showing characteristic deposits of the microtubule-associated protein tau in the form of intracellular neurofibrillary tangles in the central nervous system. Alzheimer’s disease (AD) is the most common tauopathy as well as the most prevalent neurodegenerative disorder worldwide. However, while AD patients’ brains are characterised by tauopathic neuropathology, a genetic association of AD with the *MAPT* locus has not been unequivocally established^1^,^2^,^3^,^4^,^5^. In 1998, several groups showed that mutations in the *MAPT* gene are sufficient to cause a rare familial neurodegenerative disorder termed frontotemporal dementia with parkinsonism linked to chromosome 17 (FTDP-17)^6^,^7^,^8^,^9^. The *MAPT* locus is also strongly associated with the sporadic tauopathies progressive supranuclear palsy (PSP) and corticobasal degeneration (CBD) which is likely explained by haplotype-specific regulation of alternatively-spliced exons^10^,^11^,^12^,^13^. More recently, a multitude of gene association and genome-wide association studies (GWAS) have identified *MAPT* as a highly significant genetic risk factor for sporadic Parkinson’s disease (PD), despite the lack of obvious tau tangles in the majority of PD patients^14^,^15^,^16^,^17^,^18^. More recently, imbalances in tau isoform ratios and rod-like tau aggregates have been described in Huntington’s disease^19^. In summary, the association of tau with several common neurodegenerative diseases either in the form of pathological inclusions, or as a genetic risk factor, demonstrates that tau is one of the key genes and proteins involved in neurodegeneration.

The major function of tau is the stabilization of microtubules and promotion of microtubule polymerization^20^,^21^,^22^,^23^. Other functions include the regulation of motor protein movement, the development of cell polarity, as well as modulation of N-methyl-D-aspartate (NMDA) receptor function^24^,^25^,^26^,^27^. The *MAPT* gene spans 16 exons, 3 of which - exons 2, 3 and 10 - are alternatively spliced, giving rise to six tau isoforms expressed in the adult human central nervous system^28^. Despite both intronic and exonic mutations having been identified in FTDP-17 cases, the majority of tau transgenic mouse models are based on cDNA transgene constructs of wild-type or mutant *MAPT* sequences^29^. While these models have proved to be a useful tool in elucidating the role of tau protein in neurodegeneration and its biochemical properties in the disease state, the nature of the transgene constructs limits the ability of the created mouse lines to accurately reflect all aspects of tau and its role in neurodegenerative disease.

Here we describe the generation and characterization of *MAPT* genomic DNA transgenic mouse lines expressing all six human isoforms of wild-type human tau or tau harbouring the mutation N296H from within the context of the human *MAPT* genomic locus. This mutation, which lies in the alternatively spliced exon 10 of the *MAPT* gene, has so far been described in one familial case of frontotemporal dementia with parkinsonism^30^. The case report detailed AT8-positive tau staining and accumulation of 4R tau, but no neurofibrillary tangles. Further *in vitro* experiments showed that the N296H mutation increased exon 10 inclusion and reduced microtubule assembly^31^,^32^, while there was no^32^ or a small effect^31^ on tau filament formation. We chose this particular mutation as we were intrigued by the possibility of studying a model with pathological changes heavily centered on tau isoform ratio changes rather than full-blown tangle formation. Transgenic models with these “mild”, early pathological phenotypes are highly interesting, as several studies have shown that full-blown tau tangle formation is not necessary for triggering a neurodegenerative phenotype^33^,^34^,^35^.

The transgenic lines we used for this study were generated using the PAC genomic DNA vector technology. We show that animals expressing N296H mutant tau express more 4R tau protein compared to animals expressing wild-type human tau, and display hyperphosphorylation at disease-associated tau residues in whole brain extracts of aged animals in the absence of somatodendritic accumulation of tau. We demonstrate that mice expressing mutant tau on an endogenous *Mapt*−/− background display a hyperactivity motor phenotype and increased exploration time of the open arms of an elevated plus maze compared to mice expressing wild-type human tau. Furthermore, N296H mutant mice also displayed increased immobility time in the tail suspension test. We propose that this mouse model recapitulates some of the earliest molecular phenotypes associated with frontotemporal dementia and provides an opportunity to test the normal and disease roles of human tau protein in a physiological context.

## Materials and Methods

### Breeding of animals

Male and female mice lacking microtubule associated protein tau (*Mapt−/−*)^36^ were maintained on a pure-bred C57BL6 background. The 143 kb wild-type human H1 *MAPT* locus from pBAC-*MAPT*^37^ originally from PAC61D06 (Genome Systems, St Louis, MO) was subcloned into the P1 bacteriophage-derived artificial chromosome (PAC) vector pCYPAC2 by homologous recombination gap-end joining using primers incorporating regions homologous to the *MAPT* genomic insert (lower case) and pCYPAC2 (CAPITALS): pCYPAC2_F (5′-tta agt gaa aat gta cag att gat tat ttt cac ctg gtt tct gtt aga tta tct tAA AAT CAT TTA ATT GGT GGT GCT GC-3′) and pCYPAC2_R (5′-aga tag aaa ata tca tac agc tga ctt cac tag aga gaa agt gca tca act gct tAT TGA CCC GGA ACC CTT AAT ATA AC-3′) to create pPAC-*MAPT-H1*. P1-artificial chromosomal (PAC) vectors containing a 143 kb *MAPT* transgene encoding either the wild-type *MAPT* locus^37^ or the N296H mutation, and engineered using site-directed mutagenesis and BAC recombineering methods, were prepared by CsCl double banding for microinjection into C57BL6 (*MAPT-H1*) or C57BL6/CBA (*MAPT-N296H*) mouse pronuclei. Founder pups were screened for the presence of the intact transgene by PCR and breeding lines were established. Transgenic animals were backcrossed onto a pure C57BL6 *Mapt*−/− background for a minimum of nine generations to obtain lines *MAPT-H1*+*Mapt*−/− (referred to as the H1 line) and *MAPT-N296H*+*Mapt*−/− (referred to as the N51 line). All transgenes were maintained in the hemizygous state on the homozygous *Mapt*−/− background. All animal procedures were carried out in accordance with the United Kingdom Animals (Scientific Procedures) Act (ASPA) of 1986 after approval by the University of Oxford Ethical Review Committee and the United Kingdom Home Office.

### Polymerase Chain Reaction (PCR)

PCR was carried out to determine the animals’ genotype. For genotyping, primers were used to amplify both the *Mapt* wild-type (wt) and *Mapt*−/− knock-out (KO) allele as well as the junction sequences spanning across the PAC backbone and the *MAPT* insert at the 5′ or 3′ end of the locus. Exon PCR was carried out for all *MAPT* exons to check for transgene integrity, the *MAPT* promoter, the 238 bp H1/H2 haplotype marker and the saitohin gene *STH*, which is nested between exons 9 and 10 of the *MAPT* gene. Primer sequences and respective annealing temperatures are shown in Table 1.

### Fluorescence *in-situ* hybridization (FISH)

Primary mouse fibroblasts were extracted from ear biopsies using a modified version of Kulnane *et al*.^38^. Metaphase FISH using *MAPT*-PAC DNA was performed as described previously^39^.

### RNA *in situ* hybridization

*In situ* hybridisation for transgene expression was carried out on frozen 14 μm tissue sections using a DIG-labelled LNA probe (Exiqon) designed to be specific for human *MAPT* (5′ DIG-gctcagccatcctggttcaaa-DIG 3′). Hybridisation and signal detection were carried out as previously described^40^.

### Immunohistochemistry

Mice were anaesthetized using pentobarbital and transcardially perfused with PB buffer (pH 7.4) followed by 4% paraformaldehyde (PFA). Brains were post-fixed in 4% PFA, cryoprotected in 30% sucrose and sectioned coronally to a thickness of 35 μm using a sliding microtome. Endogenous peroxidase activity was quenched by incubating sections in 3% H_2_O_2_ in TBS for 10 min. Sections were blocked for one hour in 10% normal goat serum and incubated overnight at 4 °C with antibody against human tau or tau phosphorylated at S202/T205 (HT7, AT8, Thermo Scientific). Subsequently, sections were incubated with biotinylated secondary antibody (Vectorlabs). Staining was visualized using ABC reagent (Vectorlabs) and 3,3′-diaminobenzidine. Hematoxylin was used as a counterstain.

### Western blots

Whole brain or dissected brain tissue was homogenized in ice-cold RIPA buffer (50 mM Tris, 150 mM NaCl, 0.1% SDS, 0.5% sodium deoxycholate, 1% IGEPAL CA-630, pH 7.4) supplemented with protease inhibitors (Complete, Roche) or protease and phosphatase inhibitors (PhosStop, Roche) using a tissue tearer (Biospec Products, Inc.). Protein concentrations were determined using a standard BCA assay. To assess the presence of the six human tau isoforms, proteins were dephosphorylated by incubating 80 μg of lysate with 400 units lambda protein phosphatase (New England Biolabs) at 30 °C for 45 min. Membranes were blocked in 5% milk. The following primary antibodies were used for detection: HT7, Tau-5, AT270 (Thermo Scientific), GAPDH (Abcam). Secondary antibodies used were (H + L)-horse radish peroxidase (HRP) conjugate (BioRad) or kappa light chain HRP conjugate (Abcam). ImageJ software (http://imagej.nih.gov/ij, National Institute of Health) was used for densitometric analysis of protein bands.

### Behaviour

#### Forepaw stride length

Animals were trained to walk over a sheet of white A3 paper in a straight line without running or stopping in three sessions with three trials per session. The animal’s home cage was placed on its side at the end of the paper for the mice to walk into. For testing, the animal’s forepaws were placed in black ink before the animal was allowed to walk across the paper. Forepaw stride length was measured as the mean distance between the middle toe of a footprint and the heel of the next footprint on the same side of the body for 6–7 footprints.

#### Accelerating rotarod

Mice were placed on an accelerating rotarod (ENV-577M, Med Associates), always facing the same direction. The rotarod was started and accelerated from 4–40 revolutions per minute over the course of five minutes. The latency to fall off the rod was recorded. Each animal was tested in three trials and the latency was averaged.

#### Locomotor activity

Locomotor activity in an unfamiliar environment was assessed using the San Diego Instruments Photobeam Activity System. Animals were individually housed in unfamiliar transparent cages with a thin layer of fresh bedding and without access to food or water. Ambulations over a four-hour period were recorded in 30 min intervals.

#### Stool collection

Animals were separated into individual transparent cages without bedding or access to food or water. Over a period of 60 min, faecal boli were collected in test tubes immediately after expulsion and stool frequency was recorded. The tubes were weighed before and after collection to obtain the wet weight of the stool. Samples were then dried overnight at 55 °C and weighed again to obtain the dry weight of the stool.

#### Spontaneous alternation

Animals were tested for spontaneous alternation in a T-maze paradigm to assess short-term memory. Mice were placed into the start arm facing the wall of the start arm of a black painted T maze (dimensions of arms 30 × 10 × 29 cm) and allowed to choose to enter either goal arm. The animal was trapped in the goal arm for 30 seconds after entering using a guillotine door. After 30 seconds, the animal was removed from the goal arm, the guillotine door was raised and the central partition removed. The animal was then returned to the start arm facing the end wall of the arm and allowed to choose either goal arm for the second time. Whether or not the animal alternated was recorded. The test was performed twice every day (morning and afternoon) for five consecutive days, and percentage alternation was averaged over all completed trials.

#### Elevated plus maze

Animals were placed in the centre (6 × 6 cm) of an elevated plus maze with two open and two closed arms (dimensions of each arm 35 × 6 cm) always facing the same closed arm. Animals were videotaped during a five minute test period. Videos were scored by an experimenter blinded to genotype. Animals were defined as having entered an open arm when all four paws crossed into the open arm. Percentage of time spent in the open arms over the test period was calculated.

#### Tail suspension test

Mice were individually suspended from a ring stand (distance to table ca. 30 cm) by their tails using an adhesive strip fastened ca. 1 cm from the tip of the tail. During a test period of five minutes animals were videotaped. Time spent immobile was scored by an experimenter blinded to genotype. Animals were excluded from the test if they successfully climbed their tails.

## Results

### Generation of transgenic mice expressing tau from the human genomic MAPT locus

**Figure 1:**
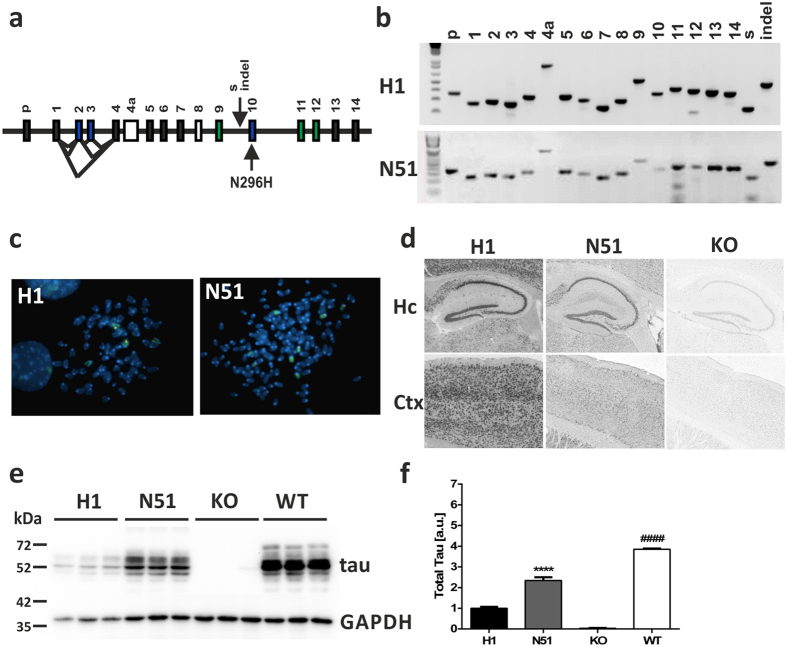
Generation of PAC-based tau transgenic mouse line expressing wild-type or N296H mutant human *MAPT*. (**a**) Exon structure of the human *MAPT* genomic locus. p - promoter, s - *Saitohin (STH*) gene, indel - 238 bp insertion relative to H2 haplotype, black exons - constitutive expression, blue exons - alternatively spliced, white exons - not present in human CNS tau protein, green exons and exon 10 - microtubule binding domain. (**b**) Exon PCR for assessment of *MAPT* transgene integrity. All 16 exons were amplified as well as the *Saitohin* gene (s) nested between exons 9 and 10 and the 238 bp insertion (indel) that characterizes the H1 haplotype. (**c**) Localization of H1 wild-type and N51 mutant transgenes with chromosome paints by fluorescence *in situ* hybridization. A single integration transgene site (red) was confirmed for both H1 (chromosome 6, green) and N51 (chromosome 9, green) line. (**d**) *MAPT* transgene mRNA expression assessed by RNA *in situ* hybridization of H1 and N51 transgenic line compared to *Mapt* KO control. Hc: hippocampus; Ctx: cortex. (**e**) Tau transgene protein expression in whole brain extract of 3–5 month old H1 and N51 animals compared to *Mapt* KO nontransgenic littermates and C57BL6 animals with endogenous tau expression. (**f**) Quantification of expression levels normalized to GAPDH loading control. One-way ANOVA followed by Bonferroni *post-hoc* correction. Results represent mean signal ± SEM. ****p < 0.0001 (compared to H1), ^####^p < 0.0001 (compared to H1 and N51), N = 3 samples per group.

The 143 kb wild-type human H1 *MAPT* locus from pBAC-*MAPT*^37^ was sub-cloned into the P1 bacteriophage-derived artificial chromosome (PAC) vector pCYPAC2 using homologous recombination gap-end joining to create pPAC-*MAPT-H1*. The vector carries the entire *MAPT* locus as well as 7.8 kb of sequence upstream of the promoter and 5.0 kb downstream of the last exon 14 (Fig. 1a). The N296H FTDP-17 mutation^30^ was engineered into the human genomic DNA pPAC-*MAPT-H1* construct using positive/negative selection/counter-selection homologous recombination in *E. coli* as previously described^41^ to generate the pPAC-*MAPT-N296H* vector. The presence of the desired point mutation was ascertained using PCR amplification and restriction enzyme digestion using *Mbo*I (Supplementary Fig. 1). The human *MAPT* locus vectors pPAC-*MAPT-H1* and pPAC-*MAPT-N296H* were purified by caesium chloride density-gradient centrifugation for pronuclear injection. The integrity of the *MAPT* transgenes in founder mice was confirmed by PCR amplification of all 16 exons, the *MAPT* promoter, the 238 bp indel insert present in the *MAPT* H1 haplotype, and the saitohin (STH) gene nested between exons 9 and 10 (Fig. 1b). Transgenic founder animals with intact transgenes were used to establish breeding lines carrying the *MAPT-H1* (referred to as the “H1” line) or the *MAPT-N296H* (referred to as the “N51” line) as a hemizygous transgene. Both lines were then backcrossed onto the *Mapt*−/− knockout maintained on a pure C57BL/6J background^36^ for at least nine generations to rule out potential confounding effects of an interaction of the human tau and the endogenous mouse tau proteins^42^,^43^. A non-transgenic *Mapt*−/− knockout (KO) group containing littermates of H1 and N51 mice was used as a control in subsequent biochemical and behavioural analyses.

We next determined the integration sites of the human *MAPT* transgene for both H1 and N51 lines by metaphase fluorescence *in situ* hybridization (Fig. 1c). The wild-type *MAPT* transgene in the H1 line was found on chromosome 6, while the *MAPT-N296H* mutant transgene in the N51 line was inserted near the centromere of chromosome 9. Both lines were shown to harbour single integration sites.

**Figure 2:**
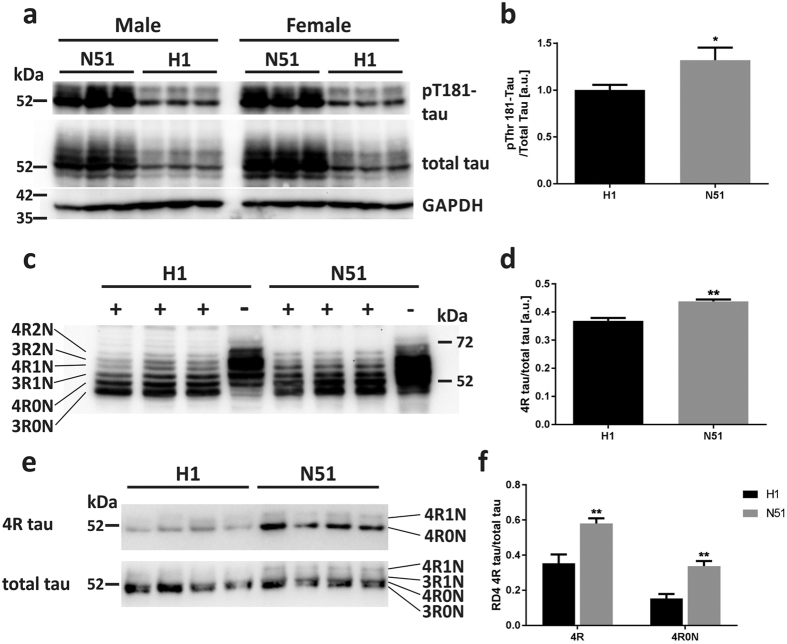
*N296H-MAPT* transgenic mice display tau hyperphosphorylation and increased expression of 4R tau isoforms. (**a**) Phosphorylation of tau at the disease-relevant T181 residue in old (18–22 months) tau transgenic whole hemispheres. Blots were probed with the phospho-T181 antibody AT270, stripped and reprobed with the pan-tau antibody A0024. (**b**) Signal quantification revealed a significant upregulation of T181 phosphorylation (N = 6 animals per genotype, one-tailed Student’s *t*-test). (**c**) Dephosphorylation of whole brain lysates revealed the presence of all six human tau isoforms in H1 and N51 animals (+dephosphorylated sample, −untreated sample) using a total tau antibody. 20 ug of H1 samples and 10 ug of N51 samples were loaded for better visualization. (**d**) Quantification of separated tau isoforms revealed a significant upregulation of 4R to total tau ratio in N51 compared to H1 animals (N = 3 animals per genotype, Student’s t test). (**e**) Detection of tau isoforms in dephosphorylated brain samples with the 4R-specific tau antibody RD4. (**f**) Quantification of 4R tau showed a significant increase of 4R tau isoforms in N51 mutant animals using the RD4 antibody (N = 4 animals per genotype, two-way ANOVA followed by Bonferroni *post-hoc* analysis). All results represent mean ± SEM. *p < 0.05, **p < 0.01.

Expression of the transgene was confirmed both by *in situ* hybridization and Western blot analysis (Fig. 1d–f). *In situ* hybridization experiments revealed high transcription levels of the transgenes compared to non-transgenic *Mapt*−/− controls (Fig. 1d) in relevant brain regions such as hippocampus and cortex. Western blot analysis of dephosphorylated lysates of whole brain demonstrated expression of all six human tau isoforms (Fig. 2c). The H1 wild-type and N51 mutant tau protein were both expressed at lower levels than endogenous mouse tau in young animals, with the human mutant N296H tau protein expressed at ~ 2-fold higher levels than the wild-type human tau protein (Fig. 1e,f).

### N296H mutant mice display increased 4R tau expression, but no tau tangle pathology

**Figure 3:**
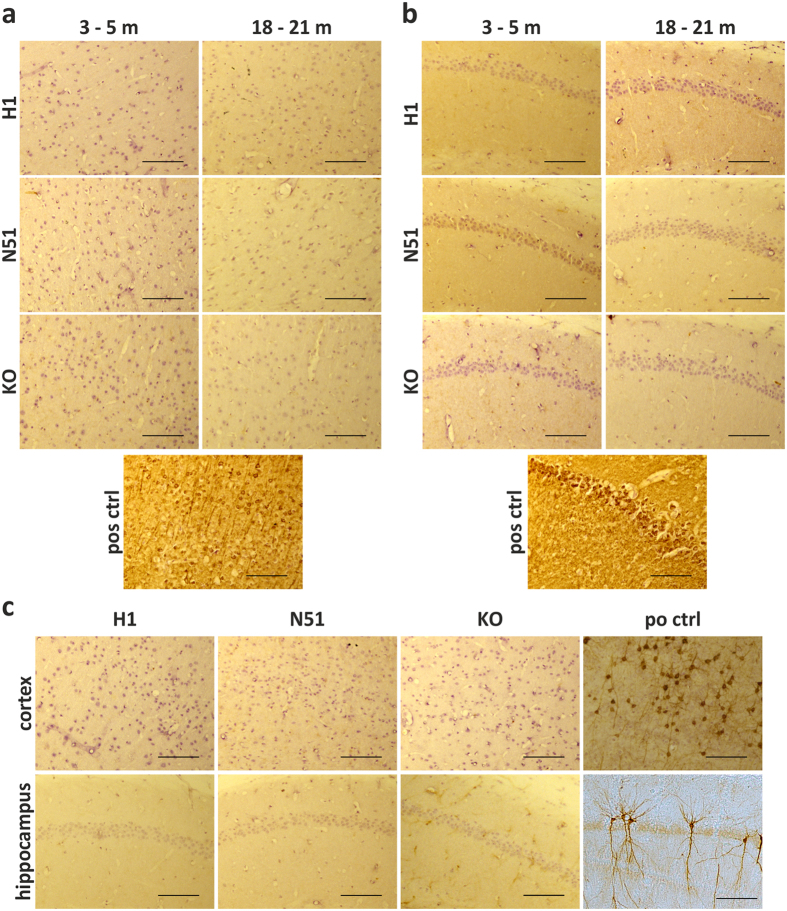
*N296H-MAPT* and *MAPT-H1* transgenic mice do not display somatodendritic accumulation of tau. Coronal sections of 3–5 and 18–21 month old animals were stained with human-specific HT7 antibody to assess the formation of somatodendritic tau inclusions at a young (3–5 months) and old (18–21 months) age. No age-related changes in staining or evidence of tau inclusion formation were found in cortex (**a**) or hippocampus (**b**) of wild-type (H1) and N296H mutant (N51) animals in comparison to their non-transgenic tau knockout (KO) littermates. (**c**) Coronal sections of 18–21 month old animals were stained with the phospho-tau antibody AT8. Both H1 wild-type and N51 mutant tau expressing lines showed no accumulation of AT8 phospho-tau compared to the knockout negative control or the P301S mutant positive control. Positive control sections (pos ctrl, obtained from P301S transgenic animal) showing heavy HT7 and AT8 staining confirm the detection of somatodendritic tau inclusions. Scale bars = 100 μm.

We carried out immunohistochemical staining of coronal sections obtained from young (3–5 months) and old (18–21 months) transgenic mice to assess the formation of intraneuronal tau inclusions in the somatodendritic compartment, a pathological hallmark of tauopathies (Fig. 3). Two different antibodies were chosen for detection of inclusions: the human-specific pan-tau antibody HT7 and the phospho-specific tau antibody AT8, which recognizes the pathological double phosphorylation of serine 202 and threonine 205^44^. We observed no age-dependent formation of somatodendritic tau inclusions in either wild-type or N296H mutant tau transgenic lines using the HT7 antibody, and only detected diffuse staining in the hippocampus in H1 and N51 sections compared to *Mapt*−/− controls. In comparison, sections obtained from a positive control P301S tau transgenic mouse line^45^ showed strong staining of somatodendritic tau in both hippocampus and cortex, demonstrating the presence of intraneuronal tau inclusions (Fig. 3a,b). Similarly, no staining with the phospho-specific AT8 antibody was detected in the cortex or hippocampus of 18–21 month old human H1 and N51 transgenic tau animals compared to tau P301S positive control sections (Fig. 3c).

Tau phosphorylation is a common pathological feature in tauopathic neurodegenerative diseases and has been shown to precede intraneuronal tau tangle formation^46^. Western blot analysis using the AT270 antibody, which recognizes tau phosphorylated at the threonine residue 181 by kinases such as GSK-3β and CDK5, was used to test the hypothesis of increased phosphorylation at the T181 site in N296H mutant tau. We observed a 32% higher level of phosphorylation at the T181 site of N296H mutant tau expressing animals compared to wild-type animals (Fig. 2a,b) (p = 0.026, Student’s *t* test). We also probed for additional phosphorylation sites using antibodies AT8 (pS202/T205), CP13 (pS202) and AT180 (pT231). However, due to a high background in the *Mapt*−/− controls and low expression of the H1 and N51 transgenes compared to wild-type endogenous tau, we could not establish satisfactory and reproducible signals above background levels for these antibodies in the transgenic lines (Supplementary Fig. S2).

The *MAPT* N296H mutation has been previously reported to cause a change in tau splicing towards increasing exon 10-containing 4R isoforms^32^. We therefore assessed the ratio of tau protein isoforms expressed in whole hemispheres of young transgenic animals (Fig. 2c,d) by Western blot analysis of dephosphorylated protein lysates. Quantification of 4R to total tau levels shows a significantly higher proportion of 4R tau in N296H mutant N51 animals compared to H1 animals (p = 0.0046, Student’s *t* test) consistent with previous reports on the function of the N296H mutation^37^ (Fig. 2d). Conversely, we detected a significant decrease in 3R tau levels compared to total tau in N296H-tau expressing animals (Supplementary Fig. S3A). In order to confirm this phenotype, we also probed dephosphorylated brain samples with a 4R isoform-specific tau antibody (Fig. 2e,f). As expected, the ratio of 4R tau to total tau was significantly higher in N296H mutant N51 animals compared to H1 animals, both when comparing total 4R tau signal and when quantifying only the most abundant 4R0N isoform (two-way ANOVA main effect of genotype F_1,12_ = 33.87, P < 0.0001; Bonferroni *post-hoc* comparison 4R: p = 0.0013; 4R0N: p = 0.0062). Conversely, the ratio of 3R to total tau was significantly lower in the N296H mutant N51 mice compared to H1 mice when probed with a 3R isoform-specific tau antibody (Supplementary Fig. S3B,C).

Overall, biochemical and immunohistochemical analysis of *MAPT*+*Mapt*−/− PAC transgenic animals revealed potential pathological features associated with early disease states, including tau hyperphosphorylation at the T181 site and increased exon 10 inclusion, without any evidence of somatodendritic tau inclusions.

### MAPT-N296H mutant mice display hyperactivity and other behavioural phenotypes

As part of a broad-based behavioural phenotyping approach we first performed tests to rule out sensory deficits which could interfere with general behavioural testing. H1 and N51 tau transgenic animals showed no deficits in responsiveness to tactile stimuli, aversive gustatory stimuli or ammonia-induced trigeminal nerve stimulation (Supplementary Fig. S4). Furthermore, no overt circadian phenotype was found in the wheel-running paradigm (Supplementary Fig. S5).

**Figure 4:**
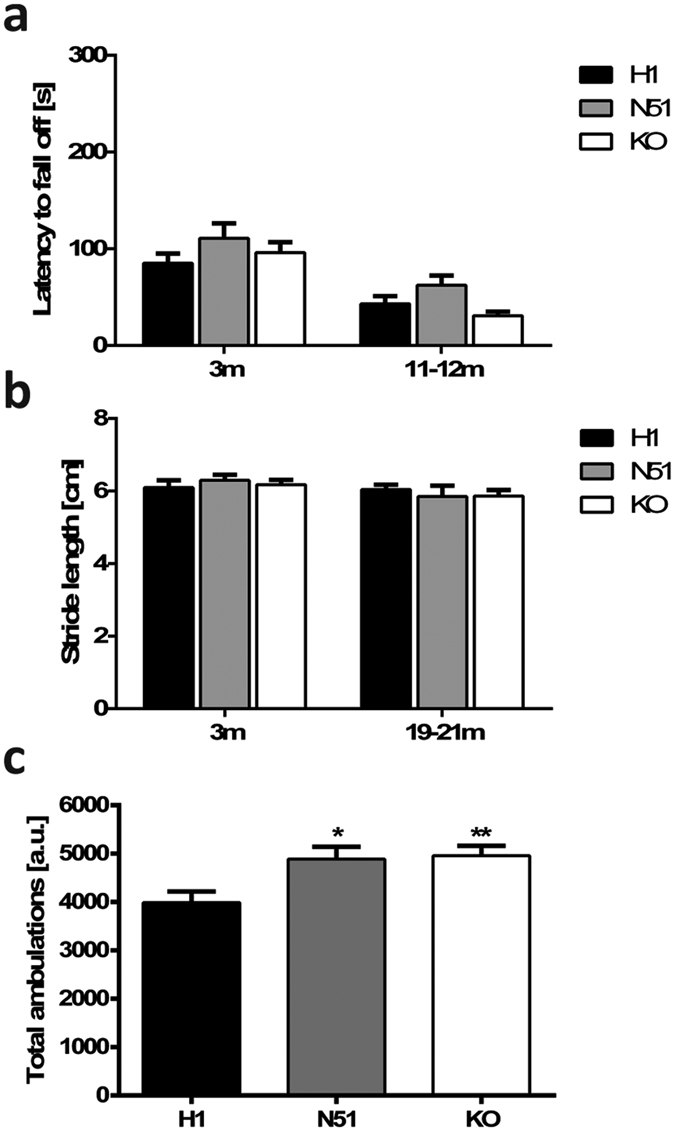
*N296H-MAPT* transgenic mice display a hyperactivity phenotype. (**a**) Accelerating rotarod performance at 3 m and 11–12 m. Animals were tested on a rotarod accelerating from 4–40 rpm over a 5 min period with three trials per day and the average latency to fall off was recorded. No significant differences between genotypes were observed at either tested time points in pairwise *post-hoc* comparisons despite a main effect of genotype. (**b**) Stride length (average distance between middle toe of a step and heel of the next step) at 3 m and 19–21 m of age. No differences in stride length between genotypes were observed in young (3 months) or old (19–21 months) mice. (**c**) Locomotor activity in an unfamiliar environment. Tau KO and N51 mutant tau expressing animals showed increased locomotor activity in 5 month old animals compared to H1 wild-type expressing animals. Results for all tests represent mean ± SEM for (**a**,**b**) N = 10–12, (**c**) N = 24–31, (**d**) N = 5–6 animals per genotype. *p < 0.05, **p < 0.01, ***p < 0.001. One-way (**c**) or two-way ANOVA (**a**,**b**,**d**) followed by Bonferroni *post-hoc* analysis.

We then performed several tests of motor function on H1 and N51 tau transgenic animals at different age points to assess the emergence of a progressive motor phenotype. While we detected a main effect of genotype in the accelerating rotarod driven by better performance of the N51 mice (two-way ANOVA, F_2,66_ = 3.168, P = 0.0486), *post-hoc* analysis did not reveal significant differences in pairwise comparisons of the genotypes (Fig. 4a). Animals were found to be too overweight at 18–20 months to perform the rotor-rod task (Supplementary Fig. S6). We measured forepaw stride length, which did not reveal any differences between wild-type and mutant tau expressing animals and their knockout littermates in young (3 months) or aged (19–21 months) mice (Fig. 4b). Locomotor activity in a novel environment was investigated over a four-hour period in transgenic mice at three different age points (5 months, 11–12 months and 19–20 months). As expected, all genotypes showed a significant age-dependent decline in locomotor activity (Supplementary Fig. S7). Interestingly, at 5 months of age both N296H tau expressing mutant N51 animals and tau knockout littermates displayed a hyperactivity phenotype in the novel environment compared to H1 wild-type human tau expressing animals in *post-hoc* pairwise comparisons (one-way ANOVA main effect of genotype F_2,77_ = 5.478, P = 0.0060) (Fig. 4c).

**Figure 5:**
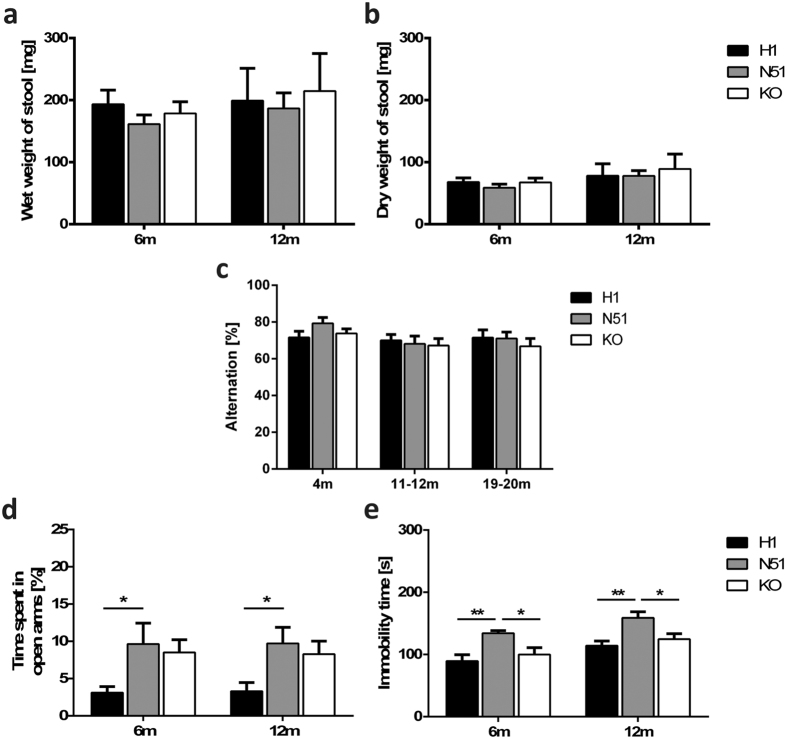
*N296H-MAPT* transgenic mice display non-motor phenotypes. (**a**,**b**) Assessment of an overt gastrointestinal phenotype revealed no differences between genotypes. (**a**) Wet weight, (**b**) dry weight of fecal boli collected over a one hour period from 6 and 12 month old animals. At both 6 months and 12 months of age, gastrointestinal function of transgenic animals was assessed by collecting fecal boli. (**c**) Spontaneous alternation in the T-maze, assessed at 4 months, 11–12 months and 19–20 months of age. No differences between genotypes were observed either in 4 month, 11–12 months or 19–20 months of age. (**d**) Elevated plus maze, assessed at 6 months and 12 months of age. At both age points, N51 mutant tau animals and tau knockout animals spent more time in the open arms of the elevated plus maze compared to H1 animals. (**e**) Tail suspension test, assessed at 6 months and 12 months of age. At both age points, N51 mutant animals displayed significantly higher immobility time compared to H1 and KO animals. Results for all tests represent mean ± SEM; for (**a**,**b**) N = 9–10, (**c**) N = 24–31 (4 m) and N = 11–13 (11–12 m, 19–20 m), (**d**,**e**) N = 10–12 animals per genotype. * p < 0.05, ** p < 0.01. Two-way ANOVA followed by Bonferroni *post-hoc* analysis.

The *MAPT* H1 and N51 animals were tested for non-motor symptoms which may model some aspects of FTDP-17, such as gastrointestinal dysfunction, cognitive impairments, sleep disturbances, anxiety and a measure of depressive-like behaviour. We investigated gastrointestinal function by collecting fecal boli of transgenic animals separated into individual cages with no access to food or water over the period of one hour. Total wet weight of stool was recorded and then the matter was dried to assess dry weight of stool. No differences between the H1, N51 and KO genotypes were observed in wet weight or dry weight of stool (Fig. 5a,b). Furthermore, stool frequencies recorded over a one hour period showed no differences between genotypes either (Supplementary Fig. S8). Taken together, these results indicate an absence of an overt gastrointestinal phenotype in the N51 animals harbouring the *MAPT* N296H mutation.

We assessed short-term spatial working memory using a spontaneous alternation paradigm in a T-maze. All animals were tested in two trials per day over the course of five consecutive days at 4 months, 11–12 months and 19–20 months of age. We observed no differences in performance in the spontaneous alternation paradigm either in 4 month, 11–12 month or 19–20 month old animals (Fig. 5c). Animals of all ages and genotypes performed significantly better than 50% (percentage of alternation expected by chance). These results suggest no indication of any gross age-dependent cognitive deficit in short-term spatial memory performance in mice carrying the *MAPT* N296H mutation.

Levels of anxiety in tau transgenic mice were assessed using the elevated plus maze test, which relies on the animals’ conflict between an aversion to open spaces versus their desire to explore novel environments. At both 6 months and 12 months of age N51 animals spent significantly more time in the open arms of the maze compared to H1 animals, possibly suggesting decreased anxiety-like behaviour (two-way ANOVA; main effect of genotype F_2,64_ = 7.349, P = 0.0013) (Fig. 5d). Tau knockout animals spent a similar amount of time in the open arms as the N51 mutant animals (Fig. 5d). While we observed no differences in the number of closed arm entries (two-way ANOVA; main effect of genotype F_2,64_ = 0.7230, P = 0.4892), we found a main effect of genotype in the number of open arm entries (two-way ANOVA; main effect of genotype F_2,64_ = 3.327, P = 0.0422). However, post-hoc pairwise comparison of genotypes yielded no significant differences (Supplementary Fig. S9).

Finally, we tested *MAPT* transgenic animals for differences in behaviour in the tail suspension test, a test used as a measure of a depressive-like state. At both tested age points (6 and 12 months), N51 animals harbouring the mutant human tau transgene showed a significantly higher immobility time compared to both H1 wild-type human tau expressing animals and *Mapt* KO animals, with the latter two showing very similar immobility times (two-way ANOVA; main effect of genotype F_2,63_ = 12.78, P < 0.0001) (Fig. 5e).

## Discussion

Our work details the generation of PAC-based transgenic mouse lines expressing the 143 kb wild-type or N296H mutant human *MAPT* locus. The lines used in our study carry transgenes covering the entire human *MAPT* locus including promoter and intronic sequences, thus enabling expression from the transgene of all six tau isoforms found in the human central nervous system. To date, most mouse models of tauopathy express tau from cDNA-based transgenes. Several of these models have been shown to recapitulate key elements of tauopathies, such as tau hyperphosphorylation, tau tangle formation, neuronal loss and various behavioural phenotypes^45^,^47^,^48^,^49^,^50^. However, imbalances in the tau isoform ratio are a defining feature of various tauopathies and several missense, silent and intronic mutations in the *MAPT* gene associated with FTDP affect splicing of exon 10, frequently leading to greater inclusion of exon 10 consistent with neuropathological findings describing deposits of exon 10^+^ (4R) tau isoforms^7^,^9^,^31^,^51^. Furthermore, it has been shown that tau isoform ratios show temporal and region-specific patterns in the brain^52^,^53^,^54^, and that different tau isoforms may serve different purposes in the central nervous system^24^,^25^,^55^. Thus, creating models capable of expressing all six human tau isoforms could provide valuable insights into the role of tau alternative splicing and its role in tauopathic neurodegenerative diseases. However, very few genomic mouse models expressing all six human *MAPT* isoforms have been described so far^42^,^43^,^56^,^57^. The first published model by Duff *et al*. was neuropathologically largely normal^43^. Only when the mouse line was backcrossed onto an endogenous *Mapt*−/− knockout background were abnormalities in tau biochemistry and neuropathology detected^42^,^56^. To rule out the confounding effects of murine tau expression, the transgenic lines in our study were bred on a *Mapt*−/− background.

Both the wild-type tau expressing H1 line and the N296H mutant tau expressing N51 line reported here were shown to harbour the entire human *MAPT* locus, including the *Saitohin* gene, which is nested between exons 9 and 10 of the tau gene. Metaphase fluorescence *in situ* hybridization confirmed single integration sites for both transgenes. Both lines express the human *MAPT* transgene at a lower level than endogenous mouse tau. Western blot analysis of dephosphorylated whole brain samples confirmed that all six human tau isoforms were expressed. In both wild-type and mutant tau expressing animals, the ratio of 4R tau isoforms - which contain the alternatively spliced exon 10 - to total tau was less than 0.5. While this expression pattern is different to the pattern in the human central nervous system, where 3R and 4R tau isoforms are expressed at equal ratios, this finding is in line with previous reports on other transgenic mouse models expressing tau from the human *MAPT* locus^42^,^43^,^57^ and may be a result of species-specific differences in the splicing machinery components. Interestingly, we demonstrated that *MAPT-N296H* mutant tau expressing mice display a significantly higher proportion of 4R tau compared to wild-type tau H1 mice. This result reflects a predominant feature of many cases of FTDP-17, and is consistent with *in vitro* studies showing increased exon 10 inclusion in splicing assays performed with N296H tau compared to wild-type tau constructs^31^,^32^.

Investigation of tau phosphorylation in our transgenic mouse lines revealed a relative tau hyperphosphorylation at T181 in whole hemisphere samples at an advanced age (18+ months) in N51 mutant animals compared to wild-type tau expressing H1 animals, in line with a tauopathic phenotype. Even at an advanced age, no somatodendritic accumulations of tau, the precursor to tau tangle formation, was observed in *MAPT* transgenic animals. The reasons for this lack of tau pathology could be the low expression levels of the mutant transgene, the choice of mutation, the short life-span of the mouse, or possibly a combination of all three. Post-mortem neuropathological assessment of a FTDP-17 patient brain with the N296H mutation revealed somatodendritic accumulation of phosphorylated tau in both glial cells and neurons; however, no neurofibrillary tangles were detected^30^. *In vitro* studies have shown no^32^ or a marginal^31^ effect on tau filament formation of the N296H mutation. Taken together this suggests that the N296H missense mutation does not drive tau aggregation to a great extent, which could explain the absence of any tau accumulation in the brains of N51 mice during their limited lifespan of two years. Alternatively, the observed low tau transgene expression levels could fail to drive the formation of somatodendritic inclusions, though several mouse models with low transgene expression levels have been described to display such inclusions^48^,^58^,^59^.

Longitudinal analyses revealed several behavioural phenotypes displayed by N296H N51 mutant tau expressing animals. While we found no gross impairment in short-term memory, as assessed by the spontaneous alternation paradigm, or in motor function assessed by stride length test and rotor-rod in animals up to 21 months, mutant tau expressing animals displayed a hyperactivity phenotype in a novel environment. Interestingly, the only case report published so far of a patient with familial FTDP carrying the N296H mutation does describes restless and aimless walking in the patient prior to eventual L-Dopa unresponsive bradykinesia and rigidity^30^. Of note, a hyperactivity phenotype has also been described in the characterization of a tau knockout mouse line^60^. At both 6 months and 12 months of age, N51 mutant tau expressing animals displayed increased exploration of the open arms in the elevated plus maze compared to H1 animals. Increased exploration of the open arms of an elevated plus maze has been described in other mouse models of tauopathy, both in the presence and absence of a hyperactivity phenotype^61^,^62^ and could be a result of decreased anxiety, potentially related to the disinhibition phenotype seen in FTDP-17 patients. However, this result may be confounded by the hyperactivity phenotype observed in N296H N51 mutant animals.

Notably, a hyperactivity phenotype and a (non-significant) increase in the open arm exploration time was observed both in the N296H N51 mutant animals and the tau knockout littermate controls, compared to wild-type human tau expressing animals. It is thus possible that these phenotypes observed in the N296H mutant mice could arise from the failure of *N296H-MAPT* to rescue the tau knockout phenotype. We observed hyperactivity and increased open arm exploration in *Mapt*−/− mice at a young age (5–6 months) consistent with the Harada tau knockout strain which displays hyperactivity and impaired fear conditioning at 10–11 weeks^60^. However, the Dawson strain, which we used as the genetic background for our transgenic lines, has not been described to show hyperactivity in young animals. It is conceivable that the C57BL6 background of our tau knockout animals may have drifted genetically from the original Dawson line, an explanation which can account for mouse phenotypes varying across laboratories.

N51 animals also displayed increased basal immobility time in the tail suspension test. The tail suspension test is used to assess depressive-like behaviour and has been shown to be sensitive to genetic variations^63^. Similar behaviour has been described in several mouse models of tauopathy^50^,^62^,^64^,^65^. Interestingly, N51 animals showed an increase in immobility time in comparison to both H1 and tau knockout mice, suggesting that this phenotype does not stem from the possible failure of N296H-tau to compensate for the loss of endogenous mouse tau described above. It is thus possible that N296H mutant tau both confers loss-of function and gain-of-function phenotypes.

Taken together, while no cognitive or motor impairment was seen in mice expressing N296H mutant tau, there was some evidence for alterations in behavioural phenotypes relevant to the only case report of a *MAPT-N296H* tauopathy patient who exhibited hyperactivity, reduced anxiety or disinhibition, and depression. Further detailed behavioural analysis will be required to fully understand the basis of the observed phenotypes, for example, to distinguish between decreased anxiety and behaviour related to hyperactivity and to elucidate the potential effects of loss and gain of tau function.

Overall, the PAC genomic DNA expression technology has allowed us to create a transgenic mouse model of tauopathy that recapitulates some aspects of frontotemporal dementia. We have shown that the *MAPT-N296H* mutation leads to an imbalance in the 3R to 4R tau splicing ratio, which is associated with tauopathic diseases, as well as tau hyperphosphorylation. Furthermore, we show that animals expressing mutant tau display relevant behavioural phenotypes, in the absence of somatodendritic inclusions. This would indicate that such inclusions are not causative of these behavioural abnormalities, but rather that these behaviours are a result of loss of normal tau function, cellular gain of function or tau isoform imbalances.

Our model is consistent with other transgenic mouse models based on imbalances in the 4R to 3R tau ratio, such as those of Dawson *et al*.^66^ and Schoch *et al*.^67^. Using an advanced mini-gene construct, Dawson *et al*. created a transgenic mouse model expressing the exon 2^+^3^+^ wild-type human 3R and 4R tau or the exon 10 splicing mutant N279K^66^. Animals expressing N279K mutant tau were shown to express more 4R tau compared to the wild-type control animals. Despite low transgene expression compared to endogenous mouse tau, which was also apparent in our own model, these mutant mice showed age-dependent immunoreactive tau inclusions, neurodegeneration and motor and cognitive deficits. We can only speculate why our own N296H model does not recapitulate some of the phenotypes described by Dawson *et al*. However, it should be noted that the N279K mutation seems to have a greater effect on exon 10 inclusion than the N296H mutation, with well over 90% of *MAPT* transcripts being 4R tau. Thus, it is an intriguing possibility that the degree of isoform shift towards 4R tau could determine the severity of the tauopathic phenotype.

The recent elegant experimental approach by Schoch *et al*. further supports the notion that increase in the 4R to 3R tau isoform ratio alone, such as we observed in N296H mutant animals, might cause tau hyperphosphorylation and behavioural abnormalities. In this study, transgenic animals expressing all six human tau isoforms were injected with isoform-switching antisense oligonucleotides (ASOs), causing a shift towards higher 4 R tau expression without altering total tau levels^67^. Compared to animals injected with scrambled ASOs, these mice showed increased tau phosphorylation, increased high-molecular weight tau species as well as abnormal burrowing behaviour and increased severity of pentylenetetrazol-induced seizures. This exciting finding lends support to the hypothesis that reduction of 4R tau by ASOs could be a therapeutic strategy for the treatment of 4R tauopathies. However, further studies will be required in order to advance this therapeutic approach. We believe that our N296H genomic mouse model might prove a valuable tool in investigating the effects of 4R tau-targeting ASO therapies.

## Additional Information

**How to cite this article:** Wobst, H. J. *et al*. Increased 4R tau expression and behavioural changes in a novel *MAPT-N296H* genomic mouse model of tauopathy. *Sci. Rep.* **7**, 43198; doi: 10.1038/srep43198 (2017).

**Publisher's note:** Springer Nature remains neutral with regard to jurisdictional claims in published maps and institutional affiliations.

## Supplementary Material

Supplementary Figures and Methods

## Figures and Tables

**Table 1:** Primer sequences used for genotyping and exon PCR.

Genotyping reaction	Forward primer	Reverse Primer	Annealing temperature [°C]
*Mapt wt*	ttg aat ctc tcc ctg gac atg g	ttg tgt caa act cct ggc gag	60
*Mapt* ko	ttg aat ctc tcc ctg gac atg g	ctt cta tcg cct tct tga	55
PAC-*MAPT* junction 5′	atg gct cat aac acc cct tg	ggt atg ggg gtc att ttt cc	63
PAC-*MAPT* junction 3′	act gac ccc acc aaa cct c	caa tga cct gac cat ttg atg	63
Promoter/Exon-1	agg aac gag ccg gga gac	gac ggc gag gca gat ttc	64
Exon 1	tga gat ctg cct gcc atg aa	cat ggc tgt cca cta acc tt	58
Exon 2	ggc tca ctg tat gtg ttc ca	agc acc agc aag caa ggc at	58
Exon 3	cag ggc tgc ttt ctg gca ta	cag cag ggc ctt gac tgc ct	58
Exon 4	cct tca ttt gct gac aca ta	caa ggc tag cat ctt tca ga	58
Exon 4A	cac cac tgc gta tct cca ca	gaa cgt cag aag cag cag ga	58
Exon 5	cag ctg gct ttc tgt gaa ca	tca tga acc tgc caa ctg ct	58
Exon 6	ctc ctc cat gtg ctg act tt	ttg caa ac caca gca gag ca	58
Exon 7	gac tct tgg tgg cag taa ct	acc tct gag agc ttc agc tt	58
Exon 8	gcc acg tga agg act cat ta	caa act gca cag gga aga ga	58
Exon 9	ctg ctg tag ctg cgc ttc ca	ctc cat gca cag tcc cac ga	58
Exon 10	ctg cca agt ccg aaa gtg ga	aga tcc tga gag ccc aag aa	58
Exon 11	gct tac aca gct gct tct ca	cac ctt gtc ttg ggc agc at	58
Exon 12	gtc ttc ttc cct cca gag ca	tcc agc cag tca aca cag aa	58
Exon 13	gat tgt gcc acc gca ctc ta	cct gat cac aaa ccc tgc tt	58
Exon 14	tgc tcc aca gaa acc ctg tt	ctg caa cca acc agg gtc at	58
Saitohin (*STH*)	ccc tgt aaa ctc tga cca cac	aca ggg aag cta ctt ccc atg	58
238 bp indel	gga aga cgt tct cac tga tct g	agg agt ctg gct tca gtc tct c	58
