# Supplementary material for: Increased 4R tau expression and behavioural changes in a novel MAPT-N296H genomic mouse model of tauopathy
Source: Sci Rep. 2017 Feb 24;7:43198. doi: 10.1038/srep43198 (PMC5324134; doi:10.1038/srep43198)
Supplement: Supplementary Figures and Methods [file srep43198-s1.doc]

***Increased 4R tau expression and behavioural changes in a novel MAPT-N296H genomic***

***mouse model of tauopathy***

Heike J Wobst^1^*, Franziska Denk^1^*, Peter L. Oliver^2^, Achilleas Livieratos^2^, Tonya N. Taylor^1^, Maria H. Knudsen^1^, Nora Bengoa-Vergniory^1^, David Bannerman^3^ and Richard Wade-Martins^1,4^

*: These authors contributed equally to this work

**Supplementary information**

**Methods**

*Adhesive dot test*

The adhesive dot test was performed to assess responsiveness to tactile stimulation. Animals were removed from their home cage and an adhesive dot (diameter ca. 8 mm) was placed on top of their head between the ears. Animals were returned to the home cage and the latency to contact or remove the dot was recorded. Cut-off for recording was set at 60 s^1,2^.

*Quinine aversion test*

The quinine aversion test was used to assess responsiveness to gustatory stimuli. Mice were removed from their home cage and habituated to individual transparent cages covered with a thin layer of bedding for 60 min before the start of the test. A cotton swab was soaked in the quinine solution (2 mg/ml). The animals were taken out of the cage and their mouths swabbed with the quinine-soaked cotton before they were placed back in the cage. The latency to groom or drag the jaw along the floor of the cage was recorded. Cut-off for recording was set at 60 s. As a control, each animal was subjected to a cotton swab soaked in water. Exposure to either quinine or water swabs was alternated between animals for counterbalance^2,3^.

*Trigeminal nerve function test*

The trigeminal nerve is one of the afferent facial nerves and relays non-odour sensations, such as nociceptive stimulation. Animals removed from their home cage and habituated to individual transparent cages covered with a thin layer of bedding for 60 min before the start of the test. For the test, mice were presented with weigh boats (4.6 x 4.6 cm) containing a drop of water or ammonia placed on either end of the cage. Time spent sniffing each weigh boat was recorded over a two minute test period. An animal was defined to be sniffing if its snout was located 1 cm or less from the edges of the weigh boat. The location of the weigh boats was counterbalanced between animals ^2^.

*Circadian screen*

Animals were individually housed in transparent cages (dimensions 44 x 26 x 12 cm, Actimetrics) fitted with running wheels, with access to food and water ad libitum. Cages were located in light controlled chambers fitted with fluorescent tubes controlled externally. Light levels were assessed with a lux meter placed at the level of the cage bottoms. Light intensity was 150 lux during light periods. Initial testing consisted of a 14 day entrainment in a regular 12:12 hour light/dark cycle (LD). Lights-on (initially at 7 am) is denoted as Zeitgeber time 0 (ZT0). Following entrainment, animals were subjected to a one hour 150 lux light pulse during the dark period of the 12:12 hour LD cycle (one day only) at ZT14 to assess negative masking, i.e. the cessation of activity during the active dark phase following an external light stimulus. Following 14 additional days of regular 12:12 hour LD, animals were subjected to a 6 hour phase advance, i.e. lights-on or ZT0 was shifted from 7 am to midnight. Following re-entrainment to the new ZT0, animals were subjected to 14 days of constant dark (DD), followed by 14 days of constant light (LL) before a return to a standard 12:12 h LD for an additional 14 days. Data analysis was carried using 10 min bins of wheel running activity using Clocklab software (*Actimetrics*).

*Antibodies*

The following antibodies were used for Western blots: Tau-5 (Thermo Scientific), Tau RD3 (1:1,000; Millipore, 05-803), Tau RD4 (1:1,000; Millipore, 05-804), AT8 (1:500; Thermo Fisher, MN1020), AT180 (1:500; Thermo Fisher, MN1040), CP13 (1:250; kind gift from P. Davies, Department of Pathology, Albert Einstein College of Medicine).


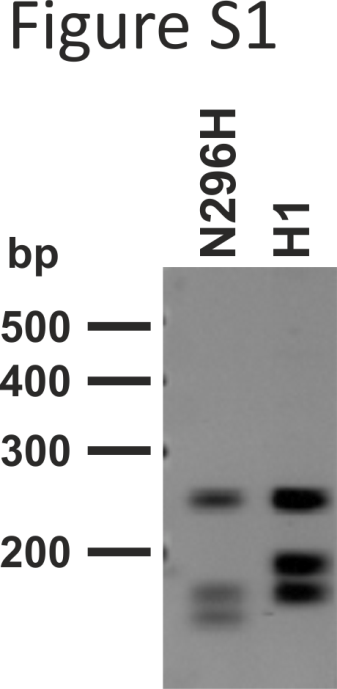


**Figure S1: Confirmation of the presence of N296H point mutation in engineered constructs.** Agarose gel of pPAC-MAPT-H1 (H1) and pPAC-MAPT-N296H (N296H) vectors digested with the restriction enzyme *MboI* to ascertain the insertion of the point mutation.


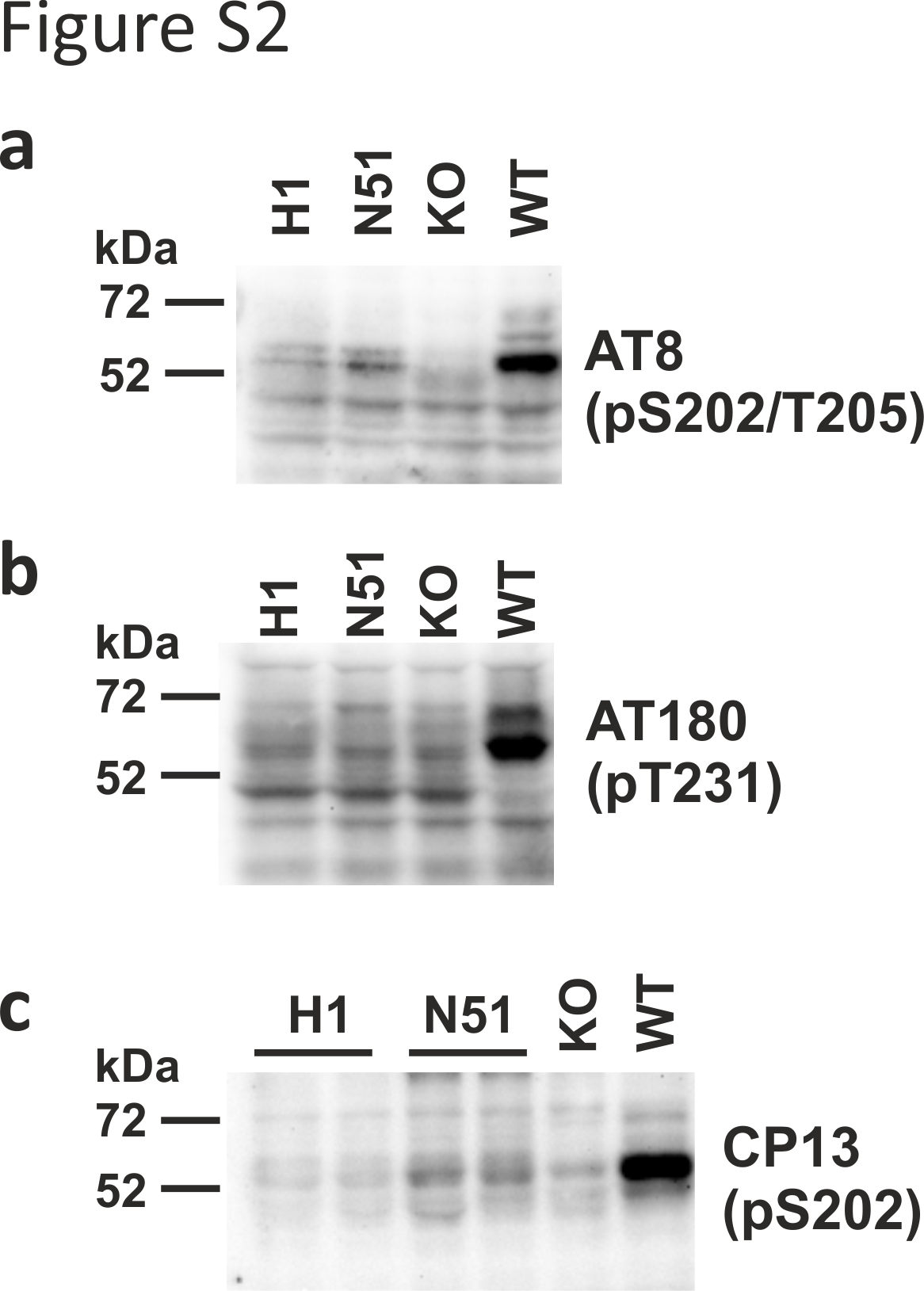


Figure S2: Detection of hyperphosphorylated tau with different phospho-antibodies. Brain lysates of aged transgenic mice (H1 and N51), *Mapt-/-* knockout control (KO) and endogenous wild-type tau expressing animal (WT). Membranes were probed with *A* AT8 (recognizing pS202/T205 site), *B* AT180 (recognizing pT231 site), *C* CP13 (recognizing S202 site) phospho-tau specific antibodies. While a clear band was picked up in the WT sample, we did not observe a quantifiable signal above background in the H1 and N51 samples. AT8 and AT180 antibodies were purchased from Thermo Fisher Scientific. CP13 antibody was a generous gift from P. Davies.


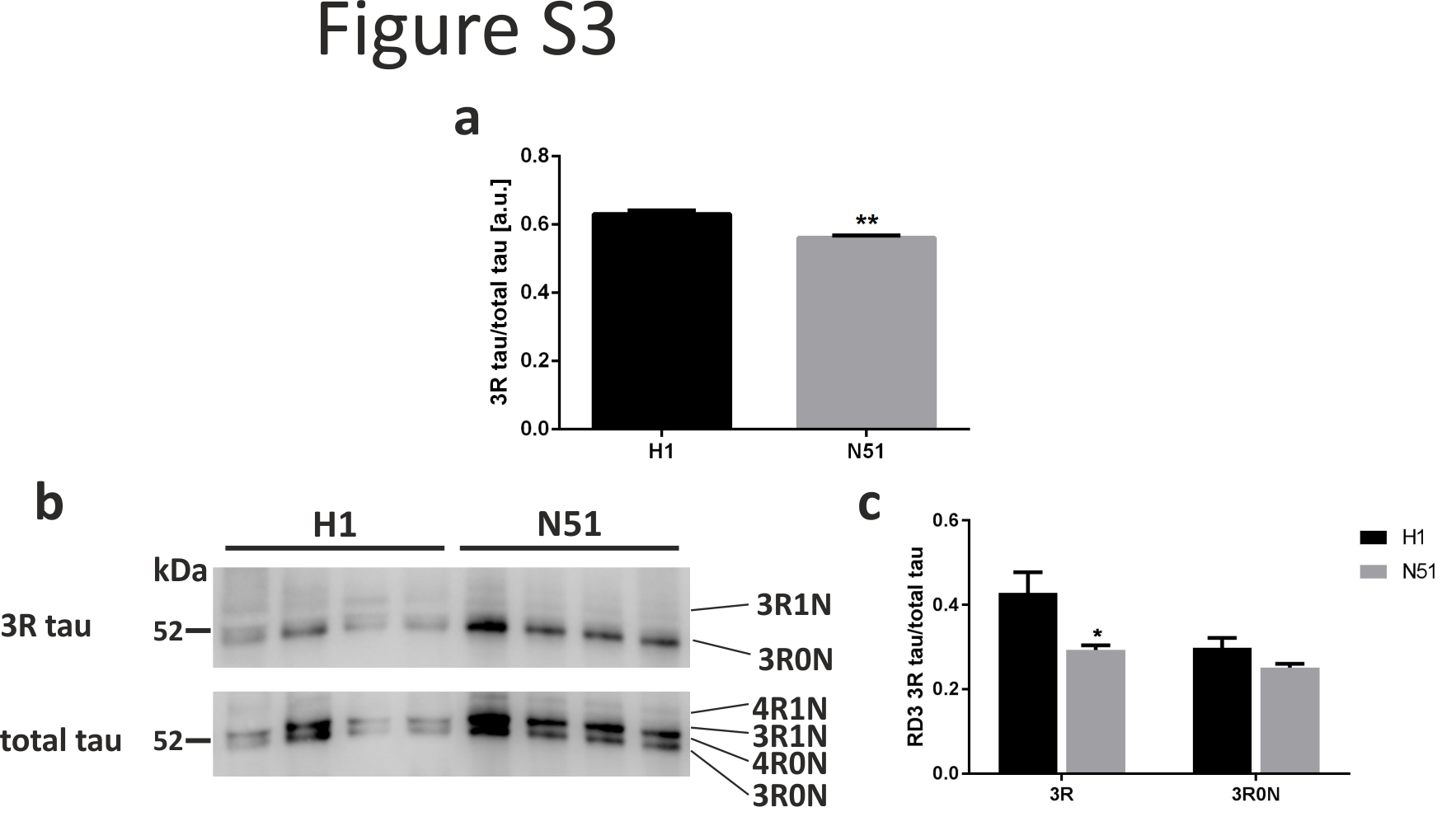


Figure S3: 3R tau expression in tau transgenic animals. *A* Expression of 3R tau in whole hemisphere lysates using a total tau antibody was normalized to total tau expression. N51 mutant tau animals showed a reduction in 3R/total tau levels (Student's *t* test). Result represent mean ± SEM for N = 3 animals. *B*, *C* Western blot (*B*) and densitometry analysis (*C*) of 3R tau isoforms in dephosphorylated samples using a 3R-specific antibody (RD3) and a total tau antibody confirmed the reduction of 3R isoforms in N51 mutant animals. Two-way ANOVA followed by Bonferroni *post-hoc* analysis. Result represent mean ± SEM for N = 4 animals. * p < 0.05, ** p < 0.01


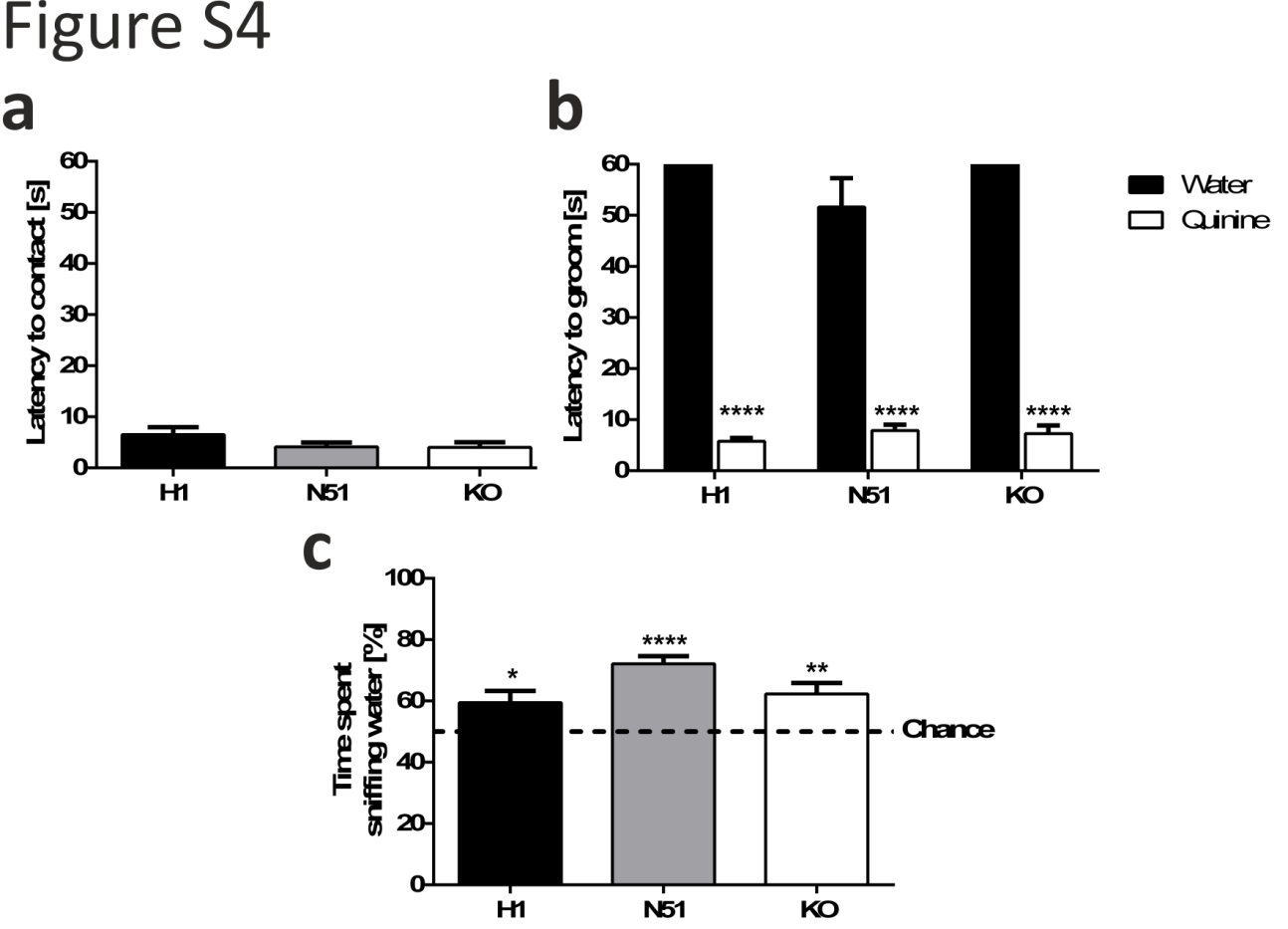


Figure S4: Assessment of general health in young animals. *A* Adhesive dot test to assess responsiveness to tactile stimulation. An adhesive dot was placed between the animals' ears and the latency to contact or remove the dot was recorded. All animals showed a fast response to the stimulus and mean latency was below 10 s for all genotypes. *B* Quinine aversion test to assess responsiveness to averse gustatory stimuli. Animals' mouths were swabbed with quinine- or water-soaked cotton swabs and the latency to groom or drag the jaw across the cage floor was recorded as an averse response. All animals showed an averse response to the bitter quinine solution independent of genotype. *C* Trigeminal nerve function test. Animals were presented with weigh boats containing a drop of water or ammonia at either end of the cage and the time spent sniffing each weigh boat was recorded. All genotypes showed a significant preference for water over ammonia, which is known to elicit an averse facial sensation through the trigeminal nerve. Asterisks represent significant differences in time spent sniffing water compared to chance levels (50%). All results represent mean ± SEM for N = 12 animals. * p < 0.05, ** p < 0.01, **** p < 0.0001, two-way ANOVA followed by Bonferroni's *post hoc* test


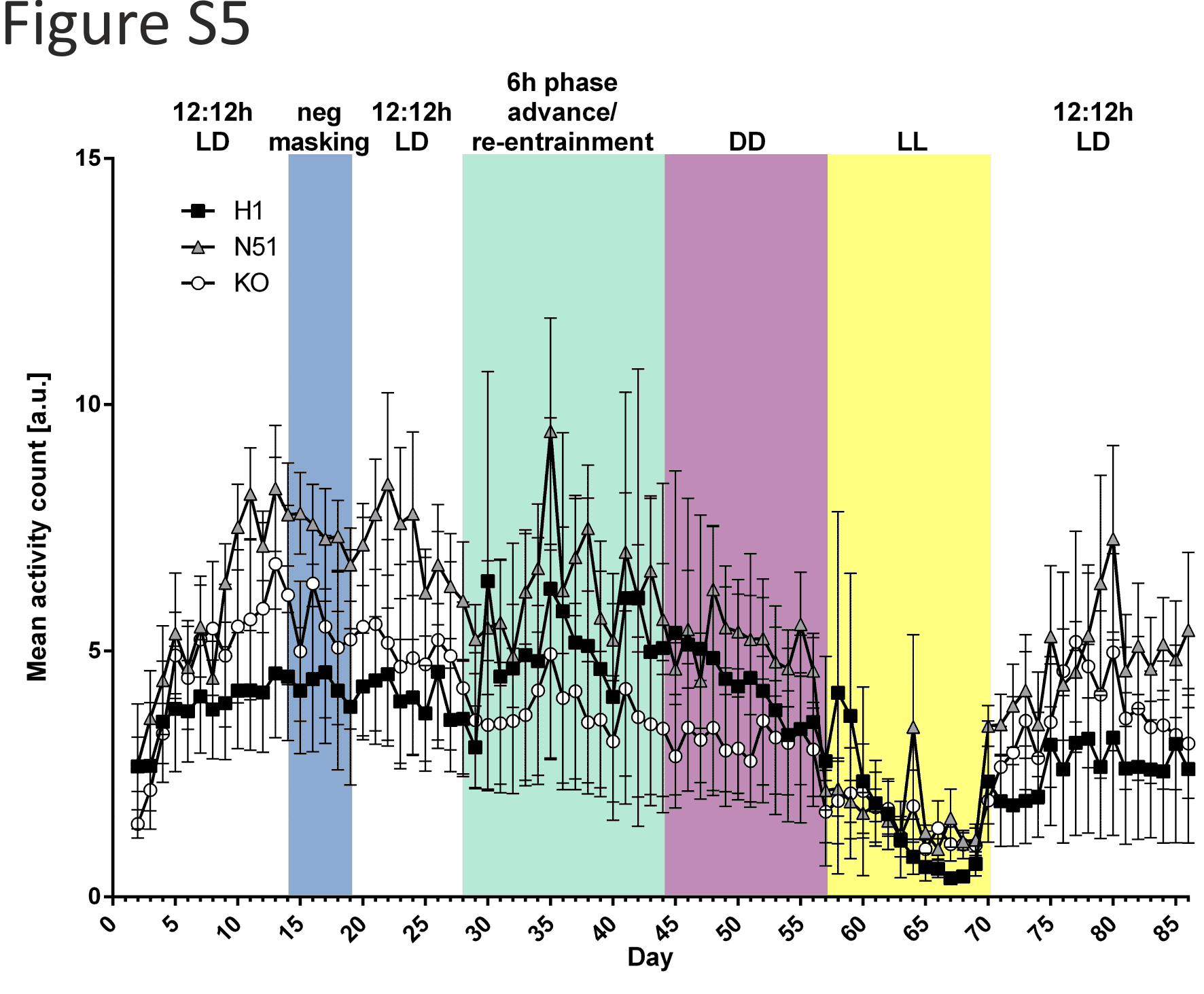


Figure S5: Mean activity profile over a 12 week circadian study. Twelve month old male H1, N51 and knockout animals were kept in cages equipped with running wheels for activity assessment during circadian screening. Animals were kept on a regular 12:12 hour light-dark cycle (LD) for two weeks, followed by a cycle where animals were subjected to a one hour light pulse during the dark cycle at ZT14 (ZT0 indicates the beginning of the 12 h light cycle) to assess negative masking. After a period of regular 12:12 hour LD, a 6 h phase advance was introduced to assess phase shift in a regular light dark cycle by tracking onset of activity. Changes on onset of activity were also assessed during a 24 h dark cycle (DD) and a 24 h light cycle (LL). All data represent mean ± SEM for N = 5 - 6 animals per genotype; two-way ANOVA.


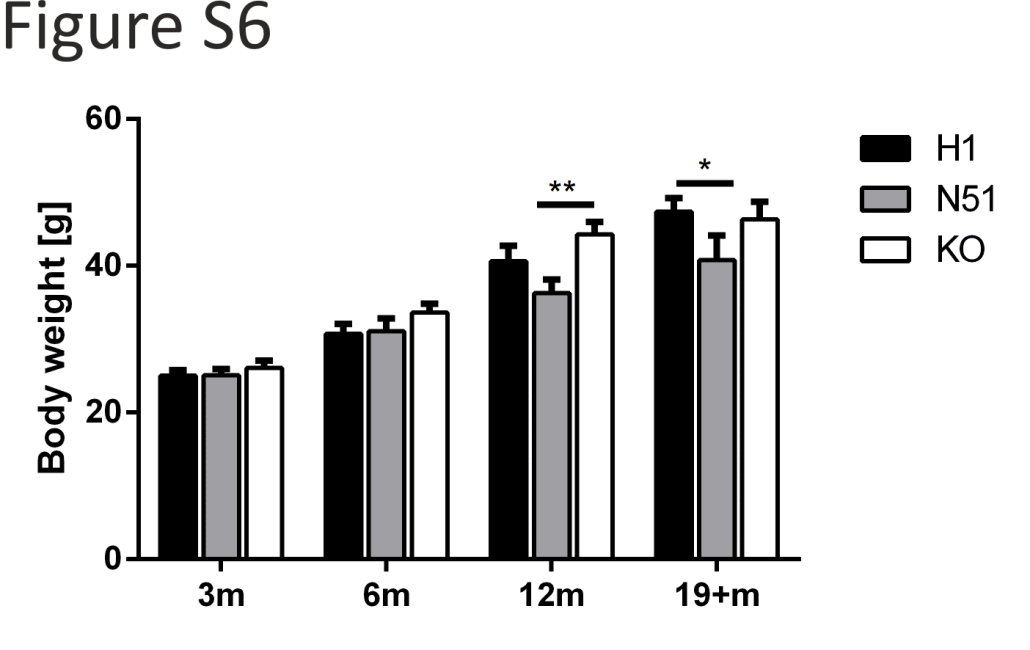


Figure S6: Body weight of tau transgenic animals. No differences between genotypes at 3 or 6 months of age. At 12 months of age, N51 animals were significantly lighter than tau KO animals, at advanced (19+ months) age lighter than H1 animals. Main effect of age F_3,127_ = 80.59, P < 0.0001; main effect of genotype F_2,127_ = 6.073, P = 0.0030. Results represent mean ± SEM for N = 9 - 16 animals. * p < 0.05, ** p < 0.01 (two-way ANOVA followed by Bonferroni *post hoc* test).


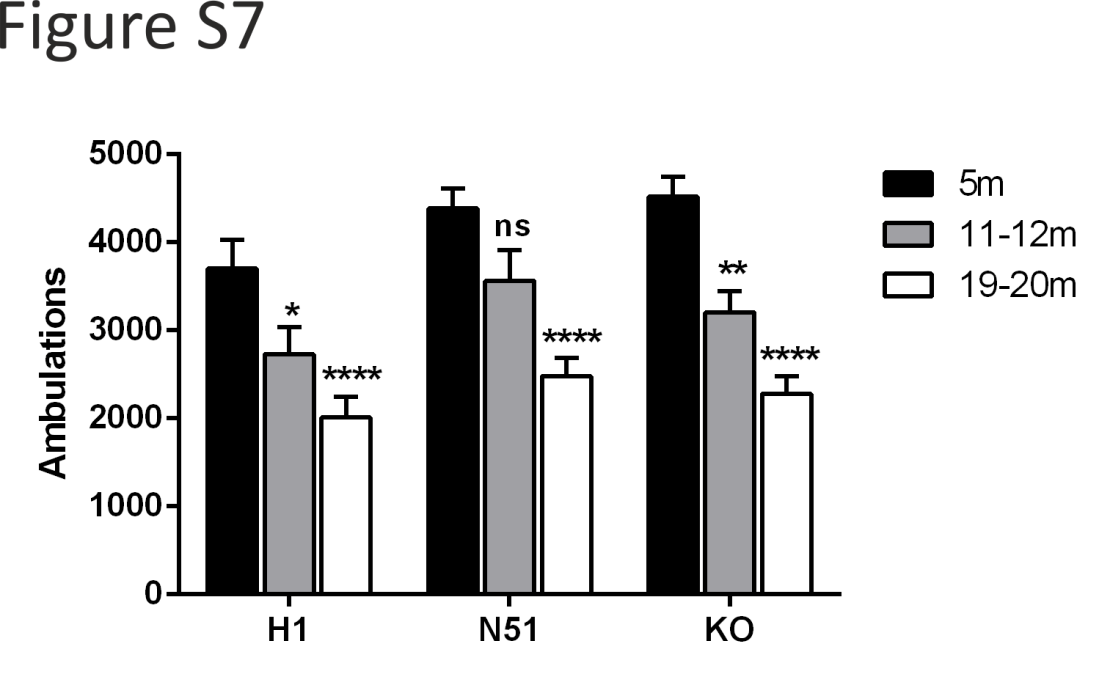


**Figure S7:** **Age-dependent decline in locomotor activity in tau transgenic and knockout animals.** Mice of all genotypes showed an age-dependent decline in locomotor activity in a novel environment (overall effect of time F_2,97_ = 40.44, P < 0.0001; overall effect of Genotype F_2,97_ = 5.293, P = 0.0066; Interaction F_4,97_ = 0.412, P = 0.7996). Results represent mean ± SEM for N = 11 - 12 animals. * p < 0.05, ** p < 0.01, **** p < 0.0001, n.s. not significant (two-way ANOVA followed by Bonferroni *post hoc* test)

**
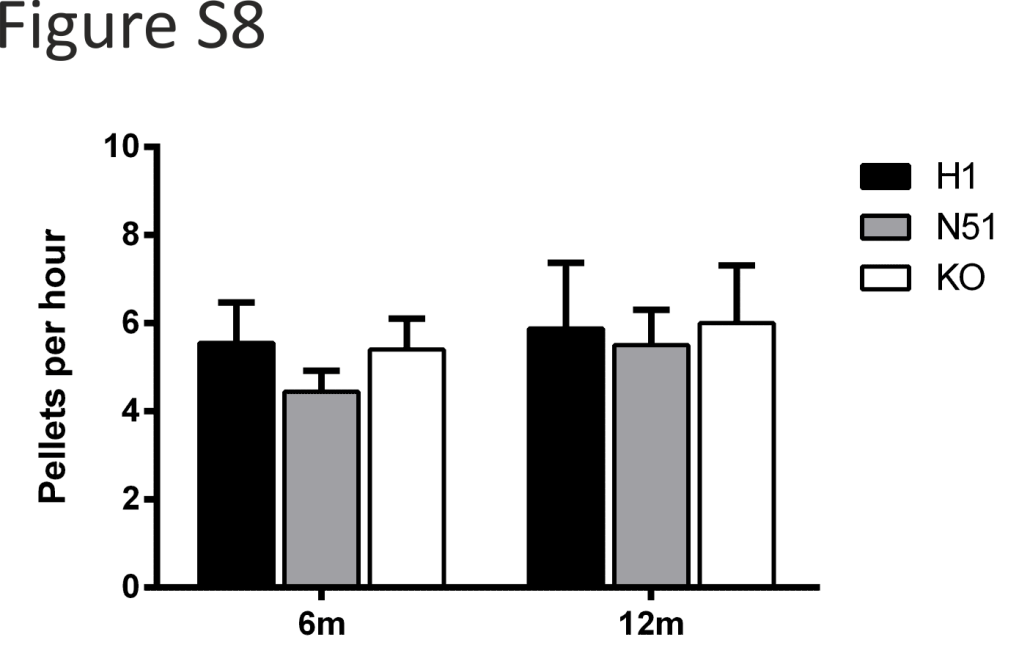
**

Figure S8: No differences in stool frequency in tau transgenic and knockout animals. Fecal boli were collected over a one hour period and expulsion frequency for each animals was recorded. Results represent mean ± SEM for N = 9 - 10 animals per genotype; two-way ANOVA followed by Bonferroni's *post hoc* test.

**
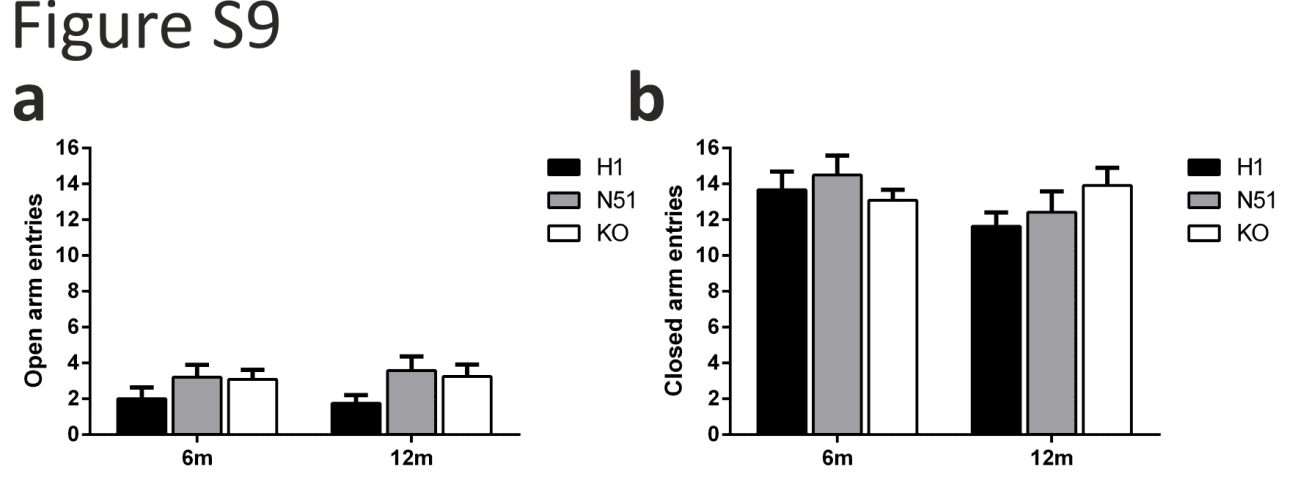
**

Figure S9: Number of A) Open and B) closed arm entries in the elevated plus maze. *A* Main effect of genotype in number of open arm entries (F_2,64_ = 3.327, P = 0.0422). *B* No main effect of genotype in number of closed arm entries (F_2,64_ = 0.7230, P = 0.4892). Pairwise *post hoc* comparison did not yield any significant differences between genotypes (two-way ANOVA followed by Bonferroni’s *post hoc* test). All results represent mean ± SEM for N = 10 - 12 animals.

**References**

1. Schallert, T., Fleming, S. M., Leasure, J. L., Tillerson, J. L. & Bland, S. T. CNS plasticity and assessment of forelimb sensorimotor outcome in unilateral rat models of stroke, cortical ablation, parkinsonism and spinal cord injury. *Neuropharmacology* **39,** 777–787 (2000).

2. Tillerson, J. L. *et al.* Olfactory discrimination deficits in mice lacking the dopamine transporter or the D2 dopamine receptor. *Behav. Brain Res.* **172,** 97–105 (2006).

3. Grill, H. J. & Norgren, R. The taste reactivity test. I. Mimetic responses to gustatory stimuli in neurologically normal rats. *Brain Res.* **143,** 263–279 (1978).
